# Chitinase 3-like 1-CD44 interaction promotes metastasis and epithelial-to-mesenchymal transition through β-catenin/Erk/Akt signaling in gastric cancer

**DOI:** 10.1186/s13046-018-0876-2

**Published:** 2018-08-30

**Authors:** Biao Geng, Jinshun Pan, Ting Zhao, Jie Ji, Chen Zhang, Ying Che, Jing Yang, Hui Shi, Juan Li, Hong Zhou, Xianmin Mu, Che Xu, Chao Wang, Yue Xu, Zheng Liu, Hao Wen, Qiang You

**Affiliations:** 1 Department of Biotherapy, Second Affiliated Hospital, Nanjing Medical University, 121 Jiangjiayuan Road, Nanjing, 210011 Jiangsu China; 2 Department of Respiratory Medicine, Yijishan Hospital, Wannan Medical College, Wuhu, Anhui China; 3 First Clinical Medical College of Nanjing Medical University, Nanjing, Jiangsu China; 4 Medical Center for Digestive Diseases, Second Affiliated Hospital, Nanjing Medical University, Nanjing, Jiangsu China; 5 Department of Thoracic Surgery, Affiliated Hospital of Nantong University, Nantong, Jiangsu China; 6 Cancer Medical Center, Second Affiliated Hospital, Nanjing Medical University, Nanjing, Jiangsu China; 7 Department of Immunology, Nanjing Medical University, Nanjing, Jiangsu China; 8 Department of Surgery, Second Affiliated Hospital, Nanjing Medical University, Nanjing, Jiangsu China; 9 Key Laboratory for Aging & Disease, Nanjing Medical University, Nanjing, Jiangsu China

**Keywords:** CHI3L1, CD44, IL-13Rα2, β-Catenin, Gastric Cancer

## Abstract

**Background:**

Enzymatically inactive chitinase-like protein CHI3L1 drives inflammatory response and promotes tumor progression. However, its role in gastric cancer (GC) tumorigenesis and metastasis has not yet been fully elucidated. We determined the significance of CHI3L1 expression in patients with GC. We also explored an as-yet unknown receptor of CHI3L1 and investigated the involved signaling in GC metastasis.

**Methods:**

CHI3L1 expression was evaluated by immunoblotting, tissue microarray-based immunohistochemistry analysis (*n* = 100), and enzyme linked immunosorbent assay (ELISA) (*n* = 150). The interactions between CD44 and CHI3L1 or Interleukin-13 receptor alpha 2 (IL-13Rα2) were analyzed by co-immunoprecipitation, immunofluorescence co-localization assay, ELISA, and bio-layer interferometry. The roles of CHI3L1/CD44 axis in GC metastasis were investigated in GC cell lines and experimental animal model by gain and loss of function.

**Results:**

CHI3L1 upregulation occurred during GC development, and positively correlated with GC invasion depth, lymph node status, and tumor staging. Mechanically, CHI3L1 binding to CD44 activated Erk and Akt, along with β-catenin signaling by phosphorylating β-catenin at Ser552 and Ser675. CD44 also interacted with IL-13Rα2 to form a complex. Notably, CD44v3 peptide and protein, but not CD44v6 peptide or CD44s protein, bound to both CHI3L1 and IL-13Rα2. Our in vivo and in vitro data further demonstrated that CHI3L1 promoted GC cell proliferation, migration, and metastasis.

**Conclusions:**

CHI3L1 binding to CD44v3 activates Erk, Akt, and β-catenin signaling, therefore enhances GC metastasis. CHI3L1 expression is a novel biomarker for the prognosis of GC, and these findings have thus identified CHI3L1/CD44 axis as a vital pathway and potential therapeutic target in GC.

**Electronic supplementary material:**

The online version of this article (10.1186/s13046-018-0876-2) contains supplementary material, which is available to authorized users.

## Background

Gastric cancer (GC) remains the second leading cause of cancer-related mortality worldwide, with the invasion and metastasis of GC constituting the major reason underlying its poor prognosis. Lymph node metastasis presents in over 50% of patients with GC when initially diagnosed, whereas peritoneum metastasis might be already present in 5% to 20% of patients undergoing gastric resection with curative intent [1]. GC develops and metastasizes as a result of the accumulation of multiple genetic and epigenetic changes. Thus, a greater understanding of how key molecular and cellular regulators drive GC invasion and metastasis is required.

Chitinase 3-like-1 (CHI3L1, also called YKL-40 in humans and BRP-39 in mice) is a member of the 18 glycosyl hydrolase gene family that contains true chitinases and chitinase-like proteins which bind to but do not degrade chitin. CHI3L1 is expressed by a vast array of cells including neutrophils, macrophages, fibroblasts, vascular smooth cells, endothelial cells, and tumor cells [2]. It has been shown that CHI3L1 plays a critical role in antipathogen, oxidant-induced, inflammation, repair and remodeling responses by regulating a variety of essential biologic processes including oxidant injury, apoptosis, pyroptosis, inflammasome activation, Th1/Th2 inflammatory balance, M2 macrophage differentiation, transforming growth factor β1 (TGF-β1) elaboration, dendritic cell accumulation and activation, and parenchymal scarring [3–6]. Recently, accumulating evidence has demonstrated that CHI3L1 enhances the inflammatory response in the tumor microenvironment and promotes tumor progression [7]. CHI3L1 is overexpressed in both serum and tumor tissue from a variety of human tumor types, such as glioblastoma, breast cancer, melanoma, gastric and colorectal cancer, for which it has been proposed as both a biomarker and potential therapeutic target [8–18]. While CHI3L1 is known to be overexpressed in GC, its significance in gastric cancer progression and metastasis is not fully elucidated. Moreover, little is known regarding the underlying mechanisms and key downstream targets of CHI3L1 in tumor metastasis.

So far the receptors of CHI3L1 include IL-13Rα2 [4], CRTH2 [16], TMEM219 [19], and galectin-3 [20]. Recent studies show that CHI3L1 binds to and signals via interleukin-13 receptor alpha 2 (IL-13Rα2) [4, 16], which was previously believed to be a decoy receptor for IL-13 as it only contains a 17 amino acid cytoplasmic tail and lacks the conserved box 1 region in signal transduction [21]. IL-13Rα2 is significantly upregulated in a number of human cancers [22–24] and has been successfully applied as therapeutic target of chimeric antigen receptor (CAR)-engineered T cells in a patient with recurrent multifocal glioblastoma [25]. Although it has been suggested that IL13Rα2-triggered activation of the FAK and PI3K/Akt/mTOR pathways was mediated by FAM120A, activation of Src family kinases and Erk1/2 were not affected by FAM120A silencing [23]. More recently, the membrane protein TMEM219 has been identified as a binding partner of IL-13Rα2. Notably, TMEM219 silencing similarly decreased CHI3L1-stimulated macrophage mitogen activated protein kinase (MAPK)/Erk and PKB/Akt activation but not Wnt/β-catenin signaling [19]. Moreover, CHI3L1 was shown to be a regulator of Th1 and cytotoxic T-lymphocyte although IL-13Rα2 mRNA was not detected in T cells [26]. These suggest that activation of the Wnt/β-catenin pathway by CHI3L1 or its regulatory signaling is mediated via an alternative mechanism, likely through an as-yet unknown receptor.

In the present study, by utilizing tissue array, immunoblotting, immunohistochemistry (IHC) analysis, and ELISA, we sought to determine the significance of CHI3L1 expression in patients with GC. Moreover, by employing co-immunoprecipitation (IP), co-localization, ELISA and biolayer interferometry (BLI), we investigated the binding and functional properties of CHI3L1 with its potential receptor. Furthermore, our in vitro and in vivo data revealed the vital roles of the CHI3L1 and its receptors in GC metastasis.

## Methods

### Human samples

A GC tissue microarray containing 100 cases of GC and paired adjacent non-cancerous tissue was purchased from Shanghai Outdo Biotech (HStmA180Su08). GC tissues and serum from patients, and the control serum from healthy adult volunteers were obtained through the Second Affiliated Hospital of Nanjing Medical University. Use of human samples was approved by the Hospital Ethics Committee.

### Mice

We utilized 6 to 8-week-old male nude or C57BL/6 J WT (Nanjing Biomedical Research Institute of Nanjing University) and CD44^−/−^ mice (on a C57BL/6 J background, Jackson Laboratories, Bar Harbor, ME) in this study. The mice were housed in a temperature-controlled environment with a 12-h light-dark cycle, and were allowed free access to water and food. All animal procedures were approved by the Laboratory Animal Core Facility of Nanjing Medical University.

### Cell lines

The AGS (Cat. #ATCC® CRL-1739™) cells was purchased from Shanghai Cafa Biological Technology Co. Ltd. (Shanghai, China). The MGC803 (Cat. #TCHu84), HGC27 (Cat. #TCHu22), BGC823 (Cat. #TCH11), SGC7901 (Cat. #TCHu46), 293 T (Cat. #GNHu17) and B16-F10 (Cat. #TCM36) cells were obtained from the Chinese Academy of Sciences (Shanghai, China). Cell lines were tested negative for mycoplasma, and authenticated by Genetic Testing Biotechnology Corporation (Suzhou, China) using short tandem repeat markers. The cells were cultured in Dulbecco’s modified Eagle’s medium supplemented with 10% fetal bovine serum, streptomycin (100 μg/ml) and penicillin (100 U/ml).

### Antibodies, proteins and peptides

The antibodies used are listed in Additional file 1: Table S1. RhCHI3L1 (Cat. #2599-CH) and recombinant mouse (rm) CHI3L1 (Cat. #2649-CH) were purchased from R&D Systems (Minneapolis, MN). RhCD44s extracellular domain (ECD) (Cat. #12211-H08H-50) and recombinant human IL-13Rα2 ECD (Cat. #10350-H08H-20) was obtained from Sino Biological (Beijing, China). CD44v3 peptide (GWEPN EENED ERDRH LSFSG SGIDD DEDFI SSTI) and CD44v6 peptide (QKEQW FGNRW HEGYR QTPKE DSHST TG) were synthesized by ChinaPeptides Co., Ltd. (Shanghai, China).

### Western blot analysis

Total proteins were prepared from gastric tissues or cultured cell samples using RIPA lysis buffers containing protease and phosphatase inhibitors from Roche Applied Science (Roswell, GA). Protein extracts were separated by sodium dodecyl sulfate-polyacrylamide gel electrophoresis (SDS-PAGE), transferred to polyvinylidene fluoride membranes (Millipore, Billerica, MA), and then probed with primary antibodies followed by incubation with appropriate secondary antibodies.

### IHC and scoring

The immunostaining index was based on the proportion of positively stained tumor cells and staining intensity. The proportion was graded as 0 (no positively stained cells), 1 (< 10%), 2 (10–50%), and 3 (> 50% of positive cells), and staining intensity was scored as 0 (no staining), 1 (light yellow), 2 (yellow brown), and 3 (brownish-yellow staining). The index was then calculated as staining intensity score multiplied by the proportion grade. Tumors with an index of 0 to 2 were considered immunostaining-low and those with an index of 3 to 9 were scored immunostaining-high.

### Co-IP assay

Cells were harvested and lysed in IP buffer (20 mM Tris pH 7.5, 150 mM NaCl, 1% TritonX-100, 1 mM ethylenediaminetetraacetic acid (EDTA), and protease inhibitors) on ice for more than 15 min. Cell lysate was centrifuged for 10 min at 12,000×g at 4°C, and the supernatant was transferred to a new tube. The supernatant was incubated with control IgG or primary antibodies, and GammaBind Plus Sepharose (GE Healthcare, Logan, UT) with gentle rocking at 4°C overnight. The next day, the pellet was washed six times with cold 1× IP buffer and then subjected to western blotting.

### RNA interference analysis

Scrambled, human CHI3L1, IL-13Rα2 and CD44 shRNAs were obtained from Shanghai Genechem Co., Ltd. (Shanghai, China) and used according to the protocols provided by the manufacturer. The cells were harvested at the indicated time points and were subjected to western blot evaluations. For genes silencing, the following target sequences were used, CHI3L1: CCGGT AGCAT CATGA CCTAC GATTT CTCGA GAAAT CGTAG GTCAT GATGC TATTT TTG. IL-13Rα2: CCGGG CTTTC GTTTG CTTGG CTATC CTCGA GGATA GCCAA GCAAA CGAAA GCTTT TTG. CD44: CCGGC GCTAT GTCCA GAAAG GAGAA CTCGA GTTCT CCTTT CTGGA CATAG CGTTT TTG.

### Immunofluorescence and co-localization assay

GC cells were cultured on poly-lysine-coated coverslips, and then fixed with 4% paraformaldehyde in phosphate-buffered saline (PBS) for 10 min. Frozen GC tissue sections were fixed in 4°C acetone. After washing by PBS, cells or frozen sections were incubated in blocking serum for 30 min and then incubated with primary antibodies at room temperature (RT) for 2 h, followed by the secondary antibody labeled with Alexa-568 for 1 h. Cell nuclei were counterstained with DAPI (Life Technologies, Carlsbad, CA). Images were acquired on a Zeiss LSM510 confocal microscope (Oberkochen, Germany).

Immunofluorescence staining in functional blocking experiments, AGS cells were cultured on poly-lysine-coated coverslips and pretreated with control antibody or CD44 neutralizing antibody (10 μg/ml) for 2 h, and incubated with or without rhCHI3L1 (500 ng/ml) for an additional 2 h. Subsequently, the cells were fixed in 4% paraformaldehyde for 10 min and permeabilized in 0.5% Triton X-100 for 5 min at RT. Then, the cells were washed three times by PBS and incubated with the primary antibody at RT for 2 h, followed by the secondary antibody labeled with Alexa-568 for 1 h. Cell nuclei were counterstained with DAPI (Life Technologies).

### Expression and purification of CD44v3 ECD

The gene encoding human CD44V3 ECD (21–310aa) was synthesized by Wuxi Qinglan Biotech. Inc. (Yixing, China), and subcloned using Hind III and Xho I restriction enzymes into the pSecTag2A vector (Invitrogen, Carlsbad, CA) in frame with the signal peptide sequence. Human 293 T cells were transfected with the above expression constructs using Lipofectamine 3000 (Invitrogen) to produce recombinant protein. The culture medium was collected after 96 h and adjusted to pH 7.5 with 10 mM HEPES. The secreted protein in the supernatant was then purified by nickel affinity chromatography, followed by dialysis against PBS buffer. Protein concentrations were measured using a BCA kit and the purity was assessed by SDS-PAGE and western blot.

### Evaluation of binding ability using a direct ELISA

CD44v3 peptide, CD44v6 peptide, rhCHI3L1 or rhIL-13Rα2 ECD was coated at 4 μg/ml in PBS buffer (100 μl per well) on High-binding microtiter plates (Corning, Armonk, NY) overnight at 4°C. After washing with PBS containing 0.1% Tween-20, the wells were blocked with 2% bovine serum albumin (BSA; 300 μl per well) in PBS for 2 h at RT. Various concentration of control BSA, rhCHI3L1, rhIL-13Rα2 ECD, or rhCD44v3 ECD were added to the plate and incubated for 1 h at RT. Following washing by PBS containing 0.1% Tween 20, anti-CHI3L1, anti-IL-13Rα2, or anti-CD44 antibody was added at appropriate proportion dilution (100 μl per well) and incubated for 1 h at RT. The assay was developed using the 1-step Turbo TMB ELISA reagent (ThermoScientific, Waltham, MA) and the absorbance was measured at 450 nm. Data represent the average of 3 independent experiments, each with triplicate wells per group.

### Kinetic binding analysis by BLI

BLI experiments were performed using an Octet K2 instrument (ForteBio, Pall Life Sciences, Port Washington, NY). Recombinant CHI3L1 or IL-13Rα2 ECD was immobilized on the Amine Reactive Second-generation (AR2G) biosensor using the amine coupling kit according to the manufacturer’s protocol. Various concentrations of the CD44v3 peptide or rhCD44s ECD were applied in the mobile phase and the association between the immobilized and flowing proteins was detected. The assays were performed in 20 mM HEPES pH 7.5, 150 mM NaCl, 0.05% (*v*/v) Tween-20, and 1 mM MgCl_2_ (or 5 mM EDTA). The binding kinetics were analyzed using ForteBio Data Analysis 9.0 software. The dissociation rate constant (K_D_) was obtained by curve fitting of the association and dissociation phases of sensograms using a heterogeneous ligand model.

### Cell proliferation assay

The effects of rhCHI3L1 on the proliferation of AGS and MGC803 cells were evaluated by Cell Counting Kit-8 (CCK8) assay. Cells were seeded at 1 × 10^4^ cells per well in serum-free medium containing various concentrations of rhCHI3L1 in a 24-well plate. The absorbance at 450 nm of each well was measured at 0, 24, 48, 72 and 96 h.

### Colony formation assay

A total of 500 GC cells (AGS or SGC7901) were seeded in 6-well plates and cultured in serum free medium containing 500 ng/ml rhCHI3L1 in the presence of control or functional CD44 neutralizing antibody (10 μg/ml, IM7, eBioscience, San Diego, CA) in 6-well plates for approximately 12 days until the majority of colonies contained more than 50 cells. Then, the colonies were fixed, stained with Giemsa solution, and counted. The clone formation efficiency was calculated as (number of colonies/number of cells inoculated) × 100%.

### EdU incorporation assay

GC cell proliferation was assessed using the EdU Assay Kit (RiboBio Inc., Guangzhou, China) per the manufacturer’s instructions.

### Cell invasion assay

AGS or MGC803 cells (1 × 10^5^) in 200 μl serum-free DMEM were seeded in a transwell apparatus (Corning Life Sciences, Lowell, MA) that was pre-coated with 60 μl Matrigel (1:3 dilution; BD Biosciences, San Jose, CA). Then, the cells were treated with rhCHI3L1 (500 ng/ml) in the presence of control or CD44 antibody for 48 h at 37°C. The cells that adhered to the lower surface were fixed with 100% methanol for 15 min at RT, and subsequently stained with crystal violet for 15 min. In each replicate, the cells were counted in six predetermined fields under a microscope. The assay was repeated at least three times independently. U0126 (Akt inhibitor), LY294002 (Erk inhibitor) and ICG001 (Wnt inhibitor) were obtained from Beyotime Biotechnology (Shanghai, China).

### Wound closure assay

AGS cells transfected with scrambled control or shRNA vector targeting CD44 were cultured to confluence in 12-well plates and scratched with a sterile pipette tip. Then, the cells were incubated in serum-free medium in the presence or absence of rhCHI3L1 (500 ng/ml). Pictures were taken either immediately (0 h) or after 24 h in culture at 37°C. Migration was quantified as a percentage of wound closure.

### Lentivirus transductions

A human CHI3L1 shRNA lentiviral vector was purchased from Shanghai Genechem Co., Ltd. and used according to the protocols provided by the manufacturer. The cells were harvested at the indicated time points and subjected to western blot assay.

### Quantification of CHI3L1 and TGF-β1 levels

Human serum CHI3L1 levels (R&D Systems; Cat. #DC3 L10) and total (R&D Systems; Cat. #MB100B) and active (Biolegend, San Diego, CA; Cat. #437707) mouse TGF-β1 levels in mouse broncho-alveolar lavage fluid were measured using ELISA kits as directed by the manufacturer.

### Assessment of melanoma lung metastasis

B16-F10 cells were administered to the mice by tail-vein injection (2 × 10^5^ cells/mouse in 200 μl DMEM) (*n* = 8 in each group). Lung melanoma metastases were quantified by counting the number of colonies that appeared as black dots on the pleural surface.

### Subcutaneous tumor growth assay

MGC803 cells stably infected with lenti-shCHI3L1 or lenti-shControl (1 × 10^7^ cells in 0.1 ml PBS) were subcutaneously injected into nude mice (6–8 weeks of age, *n* = 10 per group) per site for a total of two sites in each mouse. Tumors were measured twice weekly using calipers and the volume determined using the formula: V = (S^2^× L)/2, where V is the volume, S is the shortest diameter, and L is the longest diameter. The mice were euthanized on day 28, and the tumor size and weight were measured.

### Tumor lung metastasis assay

For lung metastasis, SGC7901 cells stably transfected with control or shRNA targeting the *CD44* gene were injected into nude mice (*n* = 7 in each group) via the tail vein at 1 × 10^6^/animal in 200 μl serum-free medium. After 6 weeks, the mice were euthanized with CO_2_, then the lungs were dissected and divided into eight parts on average. Five slides obtained from each part were used to calculate the area of metastatic lesions. The total area of invasive lesions on these slides represented the invasive tumor volume in the lungs.

### Statistical analysis

Statistical analysis was performed using GraphPad Prism software (version 7.0; La Jolla, CA) or SPSS 17.0 software (Chicago, IL). All data are presented as the means ± SEM and analyzed by ANOVA. When ANOVA was significant, post hoc testing of differences between groups was performed using the Least Significant Difference (LSD) test. A Student’s t test was used for comparing two groups. Frequencies of categorical variables were compared using the χ^2^ test. Survival curves were generated by the Kaplan-Meier method and compared by using the log-rank test. *p* < 0.05 was considered statistically significant.

## Results

### CHI3L1 upregulation in GC tissues and patient sera correlates with GC progression

**Fig. 1:**
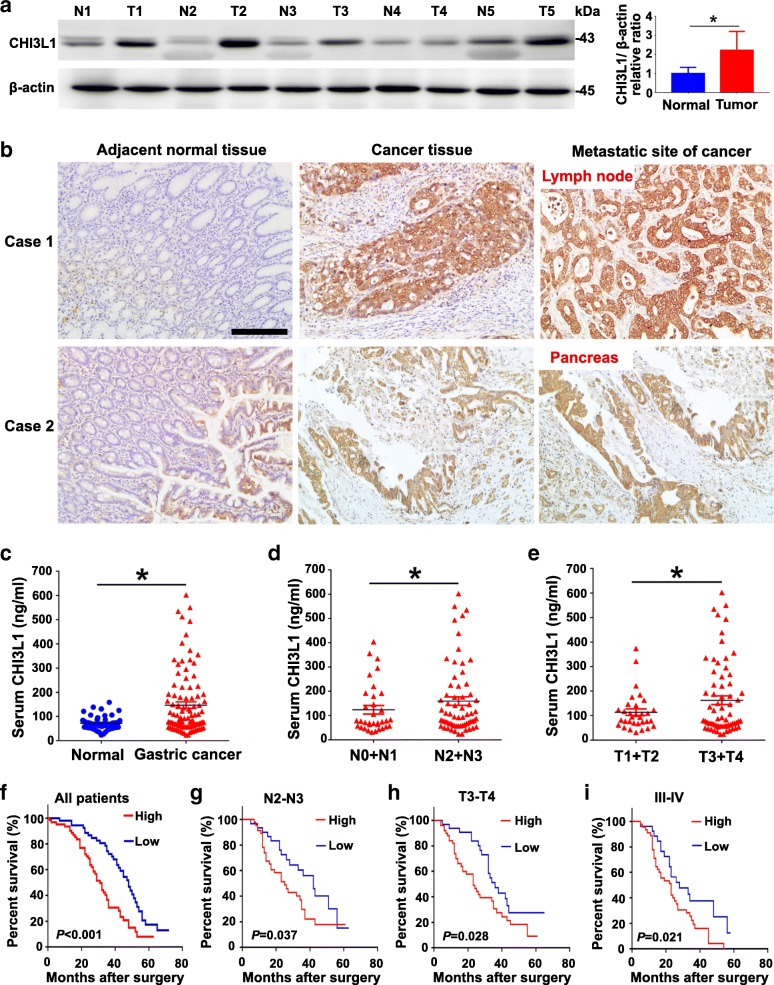
Upregulation of CHI3L1 correlates with distant metastasis and poor prognosis of human GCs. **a** Western blot analysis of CHI3L1 protein expression in 5 pairs of gastric tissues (GC and adjacent non-cancerous tissues) after curative resection. Data represent mean ± SEM; *p < 0.05 (Student’s t test). **b** Immunohistochemical analysis of CHI3L1 protein levels in GC samples on tissue microarrays. Representative examples of CHI3L1 expression in adjacent non-cancerous gastric tissues, GC tissues and GC metastases tissues (lymph node and pancreas) are shown. Scale bars represent 100 μm. **c**-**e** Serum CHI3L1 levels in GC patients (*n* = 100) and normal controls (*n* = 50) (**c**); Serum CHI3L1 levels in GC patients with different tumor invasive depth (**d**) (*n* = 17 [T1 + T2], *n* = 83 [T3 + T4]) and lymph node metastasis (**e**) (*n* = 21 [N0 + N1], *n* = 79 [N1 + N2]). Mann-Whitney U test was used to assess *p* values; *p < 0.05. **f**-**i** The overall survival of patients with high or low CHI3L1 expression in GC tissues. Kaplan-Meier test was used to analyze *p* values

To determine the significance of CHI3L1 in GC development, we first examined CHI3L1 expression in 5 GC samples using western blot analysis. CHI3L1 was significantly upregulated in GC tissues compared with adjacent non-cancerous gastric tissues (Fig. 1a). Normal gastric gland cells were almost all CHI3L1-negative or showed a mild positivity. Conversely, GC tissues and GC metastases tissues such as the lymph node and pancreas presented moderate or strong CHI3L1 expression (Fig. 1b). To further investigate the association of CHI3L1 and GC metastasis as well as the prognostic value of CHI3L1 in GCs, a tissue microarray-based immunohistochemistry study of CHI3L1 in 100 GC tissues from patients with available clinicopathological features and complete follow-up data was performed. These patients were divided into high (score 3–9) or low (score 0–2) CHI3L1 expression groups according to the immunostaining scores. Correlation analysis revealed that high expression of CHI3L1 in GC tissues was significantly associated with a more aggressive tumor phenotype (Table 1).

**Table 1:** Associations between CHI3L1 Expression and Clinical Pathological Characteristics in Patients with GC

Variable	n	Low (CHI3L1)	High(CHI3L1)	*p* value
Age				0.369
< 60	32	12	20	
≥ 60	68	32	36	
Gender				0.724
Male	64	29	35	
Female	36	15	21	
Tumor size (cm)				0.508
≤ 4	26	10	16	
> 4	74	34	40	
Differentiation				0.311
Well	42	16	26	
Poor	58	28	30	
Depth of invasion (T)				0.013
T1+ T2	15	11	4	
T3+ T4	85	33	52	
Lymph node metastasis(N)				0.024
N0 + N1	42	24	18	
N2+ N3	58	20	38	
Distant metastasis (M)				0.075
Negative (M0)	92	43	49	
Positive (M1)	8	1	7	
TNM stage				0.026
I+ II	40	23	17	
III + IV	60	21	39	

As CHI3L1 can be secreted into the serum, we further investigated CHI3L1 expression in the sera of 100 patients with GC and 50 healthy volunteers. ELISA analysis indicated that the concentrations of CHI3L1 in the serum samples from patients with GC were significantly higher than those in samples from volunteers. Notably, the increased serum CHI3L1 levels were associated with invasion depth, lymph node status, and tumor staging (Fig. 1c-e). Moreover, high expression of CHI3L1 in GC tissues was associated with a shorter overall patient survival time (Fig. 1f-i).

### CHI3L1 binds to CD44, which also interacts with IL-13Rα2

**Fig. 2:**
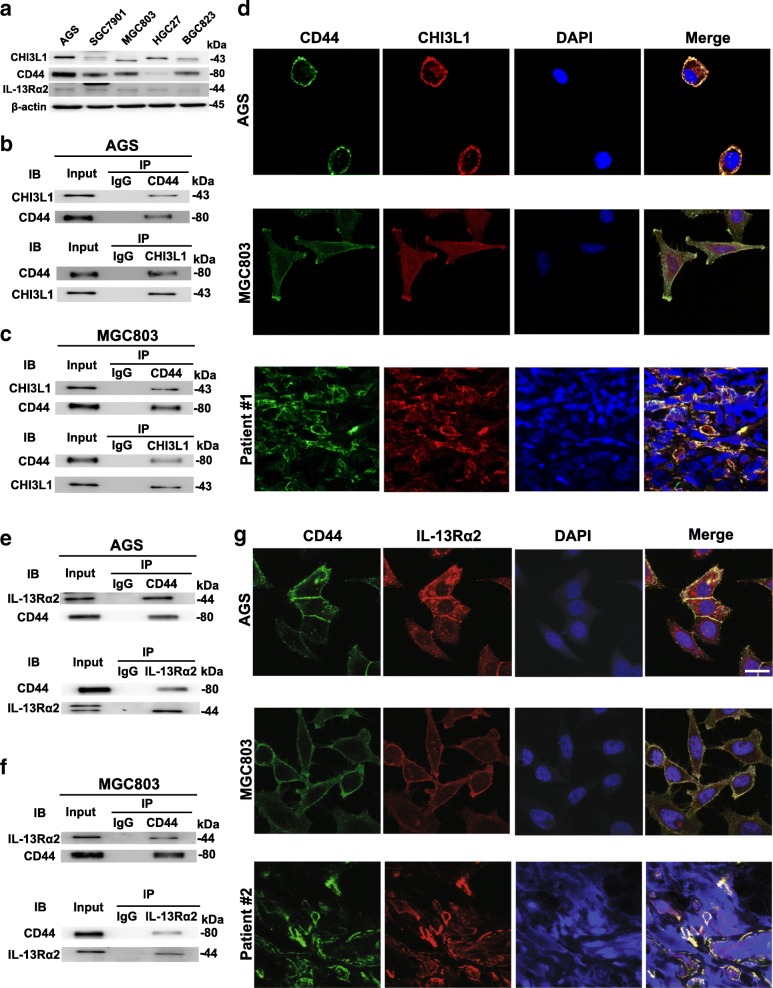
CHI3L1 binds to CD44 which also interacts with IL-13Rα2. **a** Western blot analysis of CHI3L1, CD44 and IL-13Rα2 protein expression in various gastric cancer cell lines. **b**-**c** Lysates from AGS and MGC803 cells were immunoprecipitated (IP) with control IgG and anti-CD44 or anti-CHI3L1 antibody, and then immunoblotted as indicated. Five percent of total cell lysates were used for the input. **d** Co-localization of CD44 (green) and CHI3L1 (red) in AGS (upper panel), MGC803 (middle panel) and GC tissues from patient #1 (lower panel) by immunofluorescent confocal microscopy (Magnification: 630×). Scale bars represent 10 μm. **e**-**f** Lysates from AGS and MGC803 cells were immunoprecipitated (IP) with IgG and anti-CD44 or anti-IL-13Rα2 antibody, and then immunoblotted as indicated. Five percent of total cell lysates were used for the input. **g** Co-localization of CD44 (green) and IL-13Rα2 (red) in AGS (upper panel), MGC803 (middle panel) and GC tissues from patient #2 (lower panel) by immunofluorescent confocal microscopy (Magnification: 630×). Scale bars represent 10 μm

To gain insight into the molecular mechanistic basis of the tumor facilitative effect of CHI3L1 in GC, we sought to identify CHI3L1 interacting partners. Here, we speculated that CD44 may constitute a potential receptor for CHI3L1 as they have respectively been shown to bind hyaluronic acid (HA, as its receptor) [27] and hyaluronan (as the likely preferred physiological ligand) [28]. First, we examined the expression level of CHI3L1, CD44, and IL-13Rα2 in a panel of human GC cells (Fig. 2a). The results indicated that they were expressed highly in AGS and MGC803 cells. Therefore, we performed co-IP in the lysates of these two cell lines and demonstrated the interaction between CD44 and CHI3L1 (Fig. 2b and c). Furthermore, we carried out confocal microscopy analysis to show that CD44 predominantly co-localized with CHI3L1 in GC cells (AGS and MGC803) (Fig. 2d). We also demonstrated that the interaction occurred in human primary GC tissues as well (Fig. 2d).

Recent studies show that TMEM219 binds to IL-13Rα2 complexed with CHI3L1 and mediates diverse signalling and effector responses [19]. However, both IL-13Rα2 and TMEM219 have short intracellular tails. Based on the above findings, we speculated that IL-13Rα2 interacts with CD44 to mediate signaling and biologic responses. We subsequently confirmed the association of IL-13Rα2 and CD44 by co-IP in two human GC cell lines (Fig. 2e and f). Furthermore, co-localization experiments using confocal microscopy demonstrated the interaction of IL-13Rα2 and CD44 in the plasma membrane of GC cells (Fig. 2g). In addition, IHC evaluations also indicated that IL-13Rα2 physically associated with CD44 in human primary GC tissues (Fig. 2g).

### CHI3L1 activates Erk and Akt signaling through CD44

**Fig. 3:**
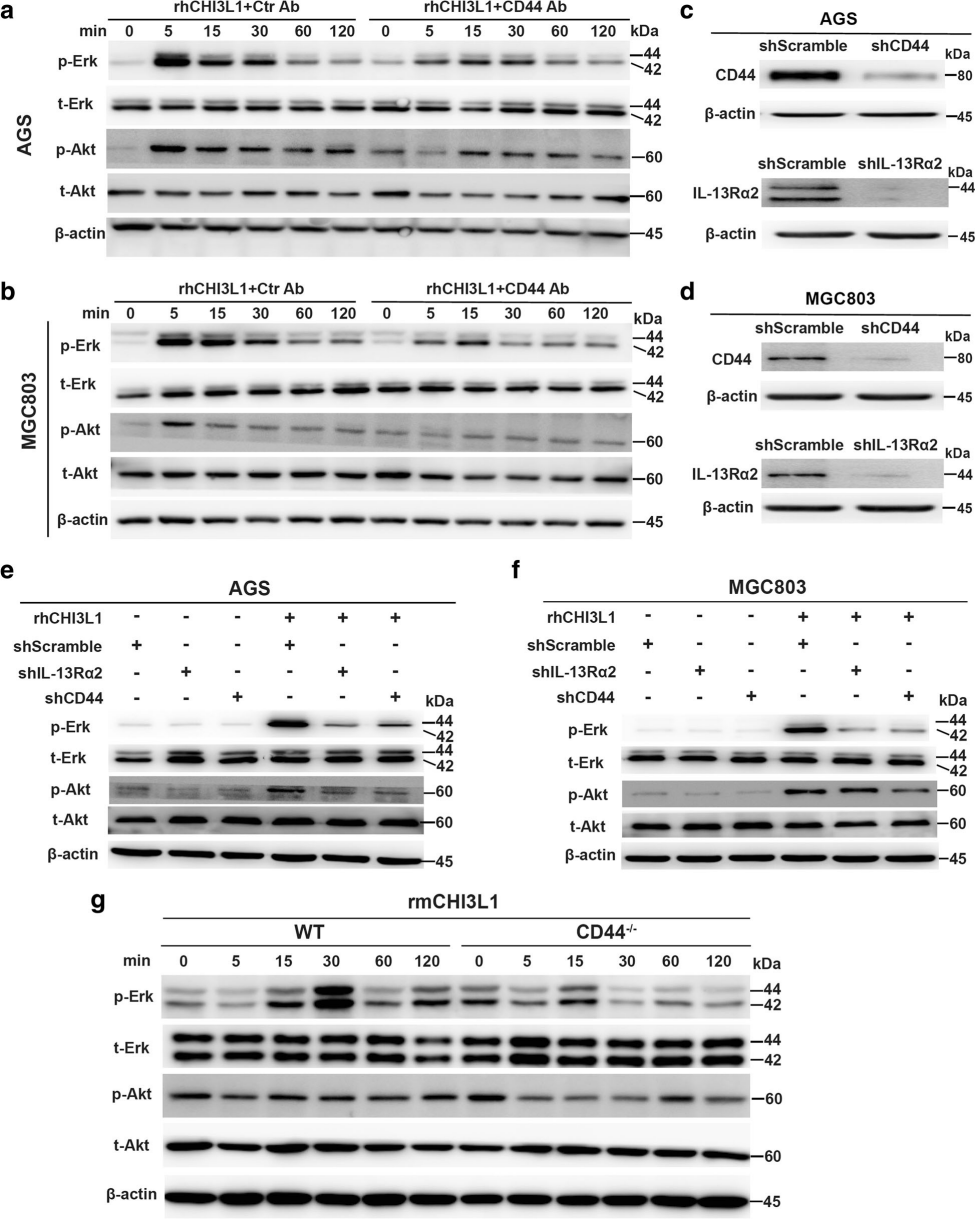
CHI3L1 triggers Erk and Akt signaling through CD44. **a** and **b** Western blot analysis of Erk and Akt activation in the AGS and MGC803 cells which were pre-exposed with the control antibody or functional CD44 blocking antibody and treated with rhCHI3L1 (500 ng/ml) for the indicated times. **c** and **d** Western blot analysis of CD44 and IL-13Rα2 knockdown efficacy in AGS (**c**) and MGC803 (**d**) transfected with scramble, CD44 or IL-13Rα2 shRNA. **e** and **f** Westen blot evaluation of the activation of Erk and Akt in AGS and MGC803 cells which stably expressing scrambled, CD44 or IL-13Rα2 shRNA and were cultured in the presence or absence of rhCHI3L1 (500 ng/ml). **g** Westen blot analysis of the activation of Erk and Akt in bone marrow-derived macrophages (BMDM) from WT and CD44^−/−^ mice which were incubated with the recombinant mouse CHI3L1 (500 ng/ml) for the noted periods of time

As CD44 closely correlates with the oncogenesis and metastasis of various cancers, we then explored the molecular mechanism of CHI3L1/CD44 signaling in GC cells. As shown in Fig. 3a and b, the expression of p-Erk1/2 and p-Akt were dramatically increased at 5 min after recombinant human (rh) CHI3L1 treatment. Notably, the elevation was significantly reduced when samples were pre-incubated with a CD44 neutralizing antibody. To further confirm these results, AGS and MGC803 cells were stably transfected with functional shRNA targeting the genes for CD44 or IL-13Rα2, and then stimulated with rhCHI3L1. ShRNA efficacy is shown in Fig. 3c and d. The data show that depletion of CD44 or IL-13Rα2 resulted in the inhibition of Erk and Akt activation in response to rhCHI3L1 (Fig. 3e and f).

Moreover, we investigated the effects of CHI3L1 on Erk1/2 and Akt activation in bone marrow derived macrophages (BMDMs) from wild-type (WT) and CD44^−/−^ mice. As can be seen in Fig. 3g, Erk activation was observed in WT BMDM cells at 15 min to 2 h after the addition of recombinant mouse CHI3L1, whereas these inductive events were significantly decreased in the cells from CD44^−/−^ mice. In contrast, no activation of Akt was observed in BMDMs from either WT or CD44^−/−^ mice.

### CHI3L1 regulates β-catenin signaling through CD44

**Fig. 4:**
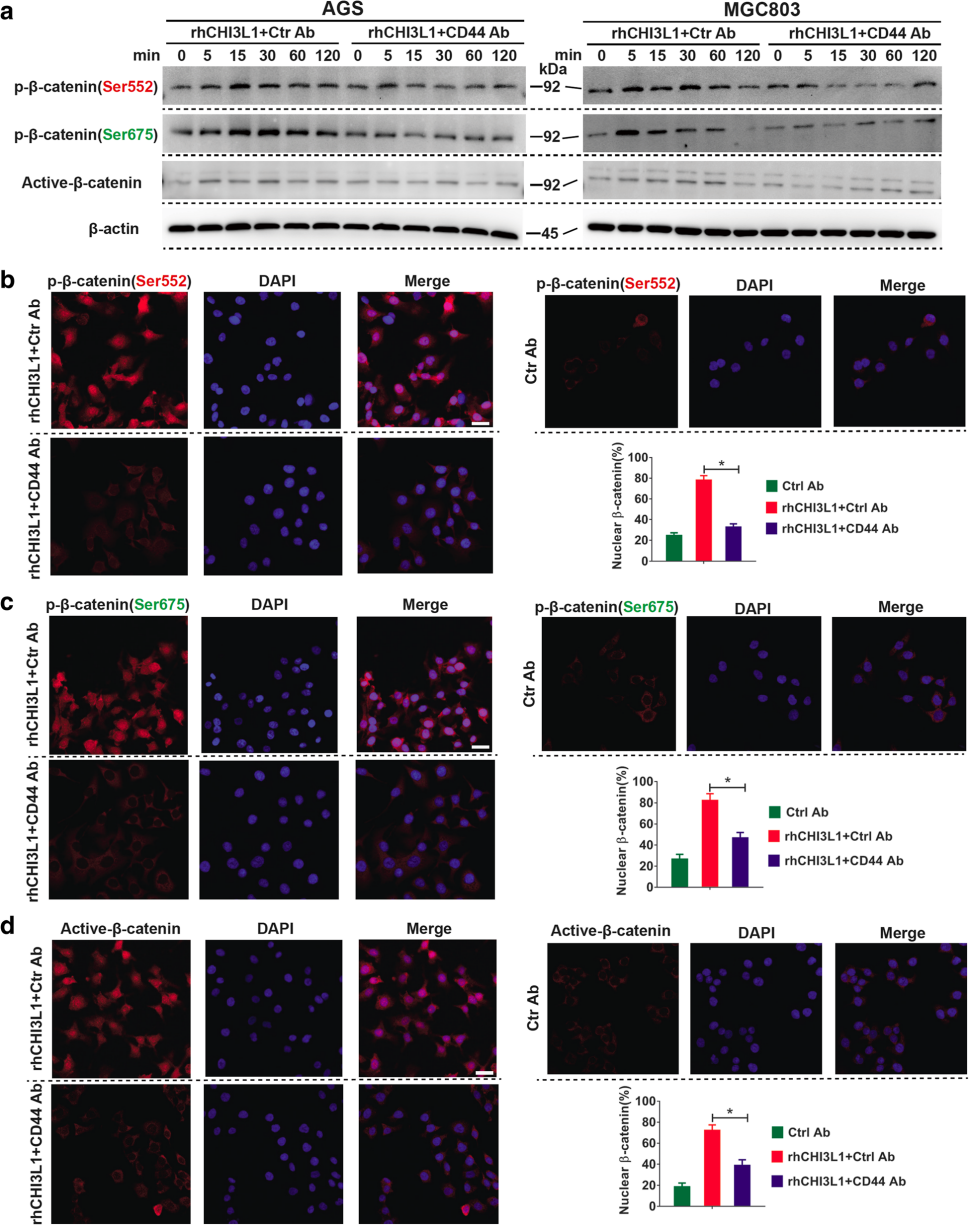
CHI3L1 regulates β-Catenin signaling via CD44. **a** Western blot analyses were used to evaluate β-catenin phosphorylation (Ser675 or Ser552), and active-β-Catenin (ABC) after exposure to rhCHI3L1 (500 ng/ml) in the presence of control or CD44 neutralizing antibody (10 μg/ml) for the noted periods of time. **b**-**d** AGS cell were pretreated with control IgG or CD44 blocking antibody (10 μg/ml). Then, the immunofluorescence assays of p-β-catenin (Ser552 or Ser675) and active-β-catenin (ABC) (all in red) were performed in AGS cell treated with rhCHI3L1 (500 ng/ml). DAPI (blue) was used as a nuclear counterstain. The quantification of nuclear β-catenin positive staining in at least 200 counted cells was presented as percentage ± SEM. Magnification: 400×, Scale bars represent 20 μm. Results shown here are the representative of three independent experiments. Statistical significance was calculated using ANOVA (**b**-**d**). *p < 0.05

Our next step was to investigate whether CHI3L1 induced the activation of Wnt/β-catenin through CD44. As shown in Fig. 4a, CHI3L1 activated β-catenin signaling by phosphorylating β-catenin at Ser552 and Ser675 in both AGS and MGC803 cells. Notably, this effect was completely inhibited by the CD44 neutralizing antibody (Fig. 4a). Then, we investigated β-catenin cellular localization following modulation of CHI3L1/CD44 axis function in AGS cells. In agreement with the western blot data, the immunofluorescence data showed a substantial increase in p-β-catenin (Ser552), p-β-catenin (Ser675), and active-β-catenin (ABC) positive staining cells upon CHI3L1 stimulation compared to control cells. Furthermore, these effects completely vanished upon pre-incubation with the CD44 neutralizing antibody. Moreover, the dramatic increase in nuclear β-catenin staining with CHI3L1 stimulation was significantly abrogated following CD44 blockage (Fig. 4b-d).

### CHI3L1 enhances GC cells growth and metastasis through CD44

**Fig. 5:**
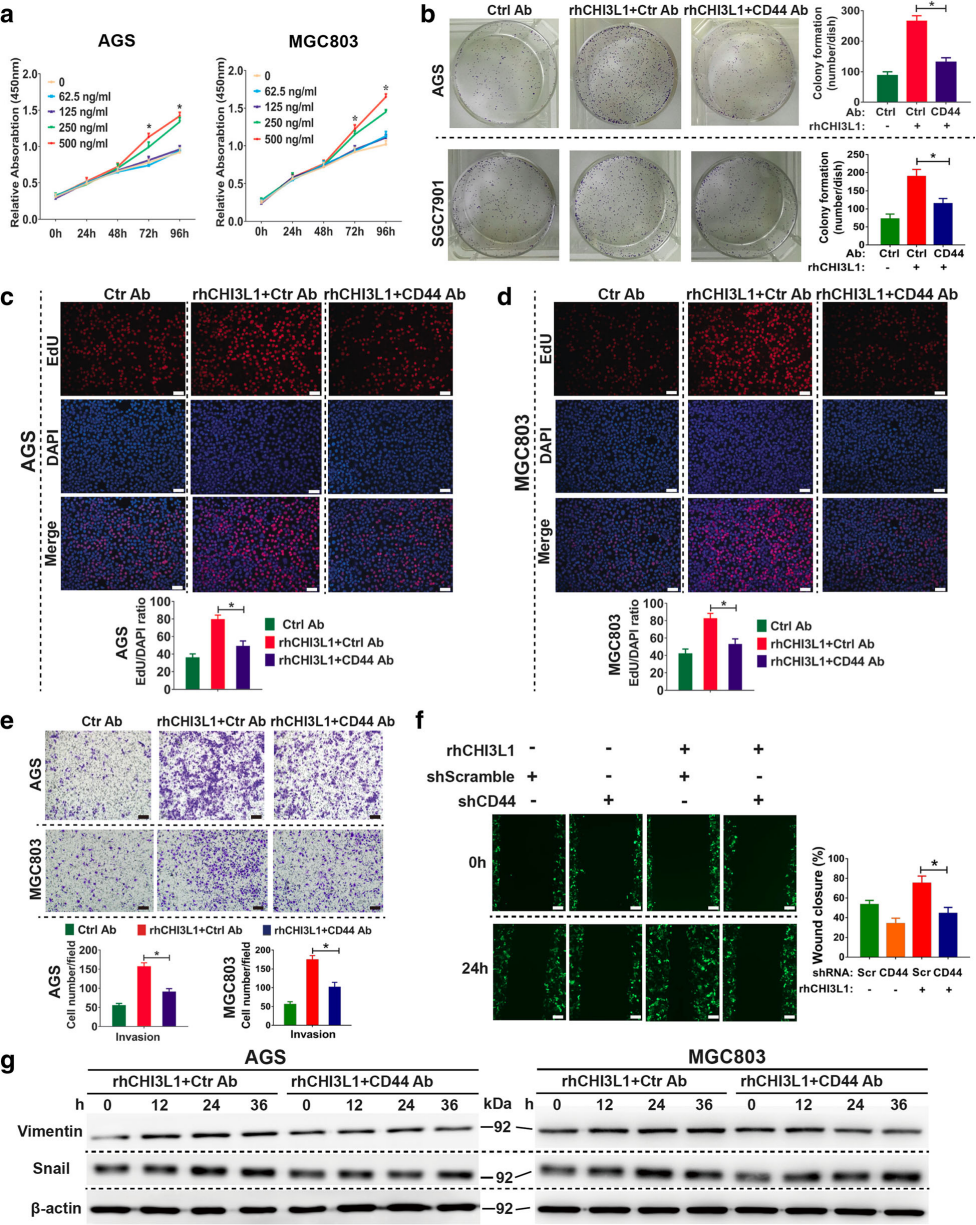
Roles of CHI3L1 in promoting GC growth and metastasis in vitro. **a** The effects of rhCHI3L1 on AGS and MGC803 cells proliferation were measured by CCK8 assay. Data are presented as mean ± SEM. **b** Colony formation assays were performed for AGS and SGC7901 cells. **c** and **d** Proliferation of AGS (**c**) and MGC803 (**d**) cells were evaluated by EdU incorporation assay. **e** The invasion assay of AGS and MGC803 cells. **f** AGS cells transfected with scramble control or shRNAs targeting CD44 were subjected to wound closure assays. **g** GC cells were incubated with rhCHI3L1for the noted periods of time. Protein levels of Vimentin and Snail were analyzed by Western blot. CHI3L1 (500 ng/ml) in combination with control or functional CD44 neutralizing antibody (10 μg/ml) were used in the experiments. Results shown here are the representative of three independent experiments. Scale bars represent 100 μm. Statistical significance was calculated using ANOVA (**a**-**f**). *p < 0.05

We subsequently evaluated the functions of the CHI3L1/CD44 axis in GC cells using in vitro cell proliferation and invasion assays. As shown in Fig. 5a, the addition of rhCHI3L1 on AGS and MGC803 cells induced cell proliferation in a dose-dependent manner. As clonal growth, the ability of a single cell to expand after colonizing on a distant site, constitutes a critical step in metastasis, we therefore examined whether CHI3L1/CD44 signaling governs this behavior. Notably, rhCHI3L1 exhibited potent ability to stimulate colony formation. However, the CD44 neutralizing antibody significantly reduced rhCHI3L1-induced clonal growth in both cell lines (Fig. 5b). The EdU proliferation assay also demonstrated that much more newly synthesized DNA could be observed in the cells with CHI3L1 stimulation, whereas the CD44 neutralizing antibody blocked this effect (Fig. 5c and d).

Our data further demonstrated that ChI3L1 significantly promoted GC cell invasion whereas the CD44 blocking antibody abrogated the effect (Fig. 5e). Moreover, knocking down the *CD44* gene in AGS cells by stably transfecting the targeting shRNA significantly inhibited cell invasion in response to rhCHI3L1 (Fig. 5f). Activation of an epithelial-to-mesenchymal transition (EMT) program has been proposed as the critical mechanism for broadly regulating invasion and metastasis by epithelial cancer cells [29]. EMT-inducing transcription factors, such as snail, facilitate E-cadherin loss, acquisition of a mesenchymal phenotype, and expression of mesenchymal markers such as vimentin [30]. Here, our data demonstrated that CHI3L1 promoted EMT by enhancing vimentin and snail expression in GC cells, whereas CD44 neutralizing antibody blocked this CHI3L1 function (Fig. 5g).

### CD44v3 physically interacts with CHI3L1 and IL-13Rα2

**Fig. 6:**
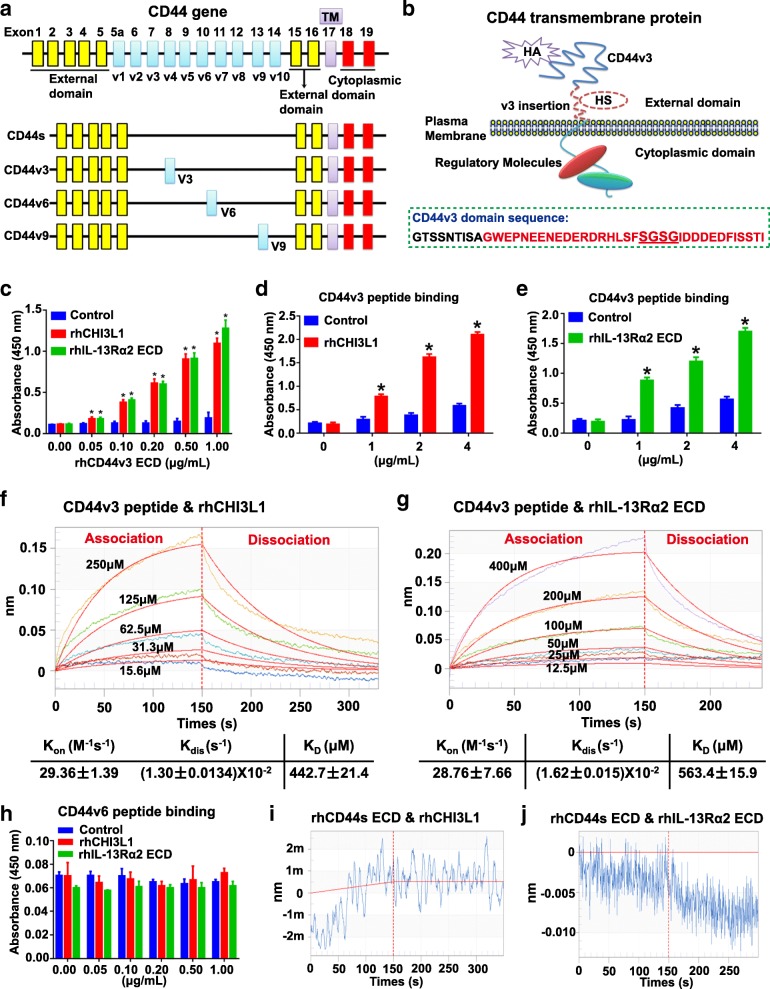
CD44v3 physically interacts with CHI3L1 and IL-13Rα2. **a** Illustration of CD44 gene and alternative spliced variants (e.g., CD44s, CD44v3, v6 and v9 isoforms) which contain external, transmembrane (TM) and intracellular domain. **b** The structure of CD44v3 transmembrane protein, which contains the hyaluronic acid (HA) binding sites at the external N-terminal region, a heparin sulfate (HS) assembly site in v3 domain, and the signaling regulator binding sites at the cytoplasmic region. CD44v3 domain amino acid sequences and v3 peptide used in the current study (in red) were listed. **c**-**e** Binding of CD44v3 extracellular domain (ECD) (**c**) or CD44v3 peptide (**d** and **e**) to rhCHI3L1 or rhIL-13Rα2 ECD. The binding affinity was evaluated by the absorbance at 450 nm in a direct ELISA. Results shown are representative of a minimum of three independent experiments. The values represent the mean ± SEM in triplicate; *p < 0.05. **f** and **g** Measurement of the binding affinity of CD44v3 peptide to rhCHI3L1 (**f**) or rhIL-13Rα2 ECD (**g**) by biolayer interferometry (BLI). Various concentrations of CD44v3 peptide were shown. All experiments were performed in triplicate. **h** Binding of CD44v6 peptide to rhCHI3L1 or rhIL-13Rα2 ECD evaluated by a direct ELISA as describe above. **i**-**j** Measurement of the binding affinity of CD44s ECD to rhCHI3L1 (**i**) or rhIL-13Rα2 ECD (**j**) by biolayer interferometry (BLI). CD44s ECD was immobilized and CHI3L1 (500 μM) or IL-13Rα2 (500 μM) was in the mobile phase

CD44 is known to undergo alternative splicing mechanisms and produces a variety of CD44 isoforms including CD44s, CD44v3, CD44v6, and CD44v9 [31] (Fig. 6a). We thus sought to explore which CD44 variant isoform was involved in its interaction with CHI3L1. CD44v3 contains an optimal Ser-Gly-Ser-Gly (SGSG) consensus motif that consitutes the only heparan sulfate (HS) assembly site among all CD44 isoforms [32–34] (Fig. 6b). Notably, CHI3L1 has been shown to bind to HS chains of the membrane-bound protein syndecan-1 in endothelial cells [15]. Therefore, we postulated that CD44v3 might act as the potential CHI3L1 binding partner. As shown in Fig. 6c, the interaction between the CD44v3 extracellular domain (ECD) and CHI3L1 was confirmed using a direct ELISA in a CD44v3 ECD concentration-dependent manner. Moreover, CD44v3 ECD was also observed to associate with IL-13Rα2 (Fig. 6c). To further confirm these findings, the peptide fragment of CD44v3 domain was administered in this ELISA binding assay, demonstrating that CD44v3 peptide binds to both CHI3L1 and IL-13Rα2, respectively, in a concentration-dependent manner (Fig. 6d and e).

In addition, a BLI analysis was performed to measure the interaction between CD44v3 peptide and CHI3L1 or IL-13Rα2. As shown in Fig. 6f, a series of concentrations of CD44v3 peptides (15.6, 31.1, 62.5, 125, and 250 μM) was used to analyze the binding affinity of CHI3L1 and CD44v3 peptide. At pH 7.4, the values were calculated as, K_on_ = 29.36 ± 1.39 M^− 1^ s^− 1^ for the association phase, K_dis_ = (1.30 ± 0.0134) × 10^− 2^ s^− 1^ for the dissociation phase, and an overall dissociation constant K_D_ = 443 ± 21.4 μM (Fig. 6f). For the binding of CD44v3 peptide to IL-13Rα2, at pH 7.4, the values were K_on_ = 28.76 ± 7.66 M^− 1^ s^− 1^ for the association phase, K_dis_ = (1.62 ± 0.015) × 10^− 2^ s^− 1^ for the dissociation phase, and an overall dissociation constant K_D_ = 563.4 ± 15.9 μM (Fig. 6g).

As it is widely accepted that CD44v6 has an important role in promoting GC progression and metastasis [35, 36], we next explored whether the CD44v6 peptide interacts with CHI3L1 or IL-13Rα2 by using a direct ELISA. As shown in Fig. 6h, there was no significant difference between the control group and the CHI3L1 or IL-13Rα2 group with respect to the absorbance at 450 nm, which suggested that the CD44v6 peptide did not bind to CHI3L1 or IL-13Rα2. To further determine whether CD44v3 ECD regions other than the v3 exon encoded a domain that might interact with CHI3L1 or IL-13Rα2, we evaluated the binding affinity of CD44s with CHI3L1 or IL-13Rα2 by using the BLI approach described above. As shown in Fig. 6i and j, no interaction was observed between CD44s ECD and CHI3L1 or IL-13Rα2.

### CHI3L1 enhances the growth and metastasis of GC in vivo

**Fig. 7:**
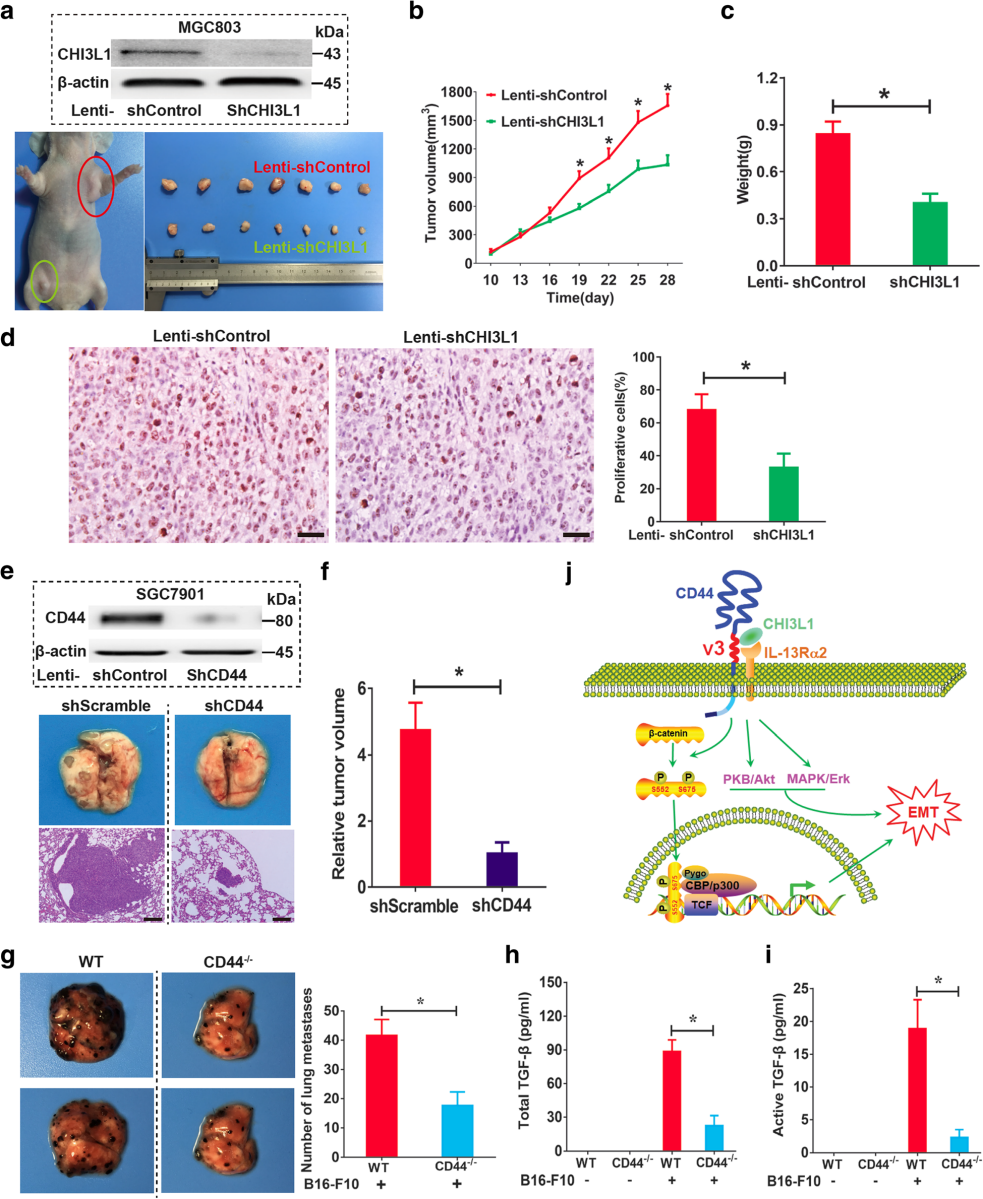
CHI3L1 mediated tumorigenesis through CD44 in vivo. **a** A representative image of tumor growth in nude mice subcutaneously inoculated with lenti-shCHI3L1- or lenti-shControl-transfected MGC803 cells. **b** The growth curve of subcutaneous tumor from MGC803 cells infected with lenti-shCHI3L1 or lenti-shControl in nude mice (*n* = 10 animals per group). **c** Comparison of tumor weight from two groups at the end of the experiment. **d** Evaluation of cell proliferative activity by Ki-67 staining in subcutaneous xenografts from MGC803 cells infected with lenti-shControl or lenti-shCHI3L1. **e** and **f** H&E staining of the representative SGC7901 cells lung metastatic lesions in nude mice (**e**). The total area of invasive lesions on the lung slice section represents the invasive tumor volume in the lungs (**f**). Sizing bar indicates 100 μm. **g** Representative photograph of lungs from WT and CD44^−/−^ mice 2 weeks after challenge with B16-F10 melanoma cells. Comparison of the number of pleural melanoma colonies in lungs from tumor cells challenged WT and CD44^−/−^ mice (*n* = 8 animals per group). **h** and **i** The levels of total (**h**) and active (**i**) TGF-β1 in broncho-alveolar lavage (BAL) fluids from WT mice and CD44^−/−^ mice. **j** Schematic representation of the CHI3L1/CD44-IL-13Rα2 signaling pathway in promoting GC cell metastasis. Data are presented as mean ± SEM, *p < 0.05 (Student’s *t* test)

To explore the role of CHI3L1 in tumor metastasis, we further utilized xenograft tumor models by subcutaneously injecting MGC803 cells transfected with lenti-shCHI3L1 or lenti-shControl into nude mice. Subcutaneous tumor growth was then monitored and compared between the two groups. As shown in Fig. 7a and b, the growth volume of the tumors developed by the lenti-shCHI3L1-transfected cells was significantly suppressed compared with that of the control group. Accordingly, the net weight of the corresponding tumors was also significantly reduced compared with control weights at termination of the experiment (Fig. 7c). In concordance with the in vitro findings, significantly fewer proliferating cells were observed in the xenografts from lenti-shCHI3L1-transfected cells, as indicated by Ki-67 assay (Fig. 7d). To determine whether the CHI3L1/CD44 axis governs GC cell metastasis in vivo, we evaluated experimental lung metastasis after lateral tail vein injection of tumor cells. The results demonstrated that stable knockdown of the *CD44* gene in SGC7901-M cells strongly suppressed lung metastasis in this model (Fig. 7e and f).

### Contribution of CD44 to melanoma metastasis and TGF-β1 production in vivo

Recent studies have shown that the metastasis of malignant melanocytes to the lung is mediated by an IL-13Rα2-dependent mechanism, which requires the production of TGF-β1 [4, 14, 37]. As described above, our data have demonstrated that CD44 physically interacts with IL-13Rα2. It has been also shown that CD44 forms a complex with TGF-β1 receptor I (TGF-βRI) and enhances its activity [38, 39]. Thus, we next examined the roles of CD44 in these responses by comparing the metastasis of B16-F10 melanoma cells and TGF-β1 production in WT and CD44^−/−^ mice. Melanoma cell administration caused massive lung metastasis in WT mice whereas this metastatic response was markedly decreased in lungs from CD44^−/−^ mice (Fig. 7g). Notably, melanoma metastasis was associated with significant increases in the levels of total and activated TGF-β1 in lungs from WT mice (Fig. 7h and i). Accordingly, both TGF-β1 levels were significantly decreased in CD44^−/−^ mice. When considered in combination, these results demonstrate that endogenous CD44, similar to IL-13Rα2, plays a critical role in pulmonary melanoma metastasis and TGF-β1 production. As summarized in the schematic diagram in Fig. 7j, our data demonstrated that CHI3L1 binds to CD44, which also interacts with IL-13Rα2, and therefore promotes GC development and metastasis through regulation of the Erk and Akt, as well as β-catenin signaling pathways.

## Discussion

Understanding the molecular basis of gastric tumorigenesis is a crucial step for the development of novel anticancer therapeutic approaches. In this study, we show that CHI3L1 is commonly upregulated in the serum and cancer tissue of patients with GC. We further investigated the clinical importance of CHI3L1 in 100 patients with GC and found that CHI3L1 positively correlated with GC progression. Moreover, the overall survival of patients with high-level CHI3L1 expression was significantly shorter than that of other patients with GC exhibiting low-level CHI3L1 expression as assessed by Kaplan-Meier survival curve analysis. In addition, we evaluated the prognostic significance of CHI3L1 expression according to different clinicopathological factors, and found that significant differences were observed in patients with T3-T4, and lymph node metastasis. These data indicated that CHI3L1 was significantly associated with shorter survival of patients with a more aggressive tumor phenotype, which suggests that CHI3L1 may serve as a new, valuable prognostic marker for patients with GC. Interestingly, our data show that CHI3L1 upregulation correlated with GC aggressiveness, not differentiation (Table 1). Cell differentiation results from the regulation of gene expression, which is mainly involved in epigenetic control. The degree of differentiation is an important reference data for cancer diagnosis and treatment, but the aggressiveness of cancer cells needs to be comprehensively judged by the combination with invasion depth, lymph node status, and tumor staging. These indicated that CHI3L1 was mainly involved in the process of cancer metastasis.

Metastasis constitutes a major hallmark of cancer, and comprises a complex multistep process involving alterations in the dissemination, invasion, survival, and growth of new cancer cell colonies. To better understand the function of CHI3L1 in GC metastasis, we sought to find the receptor of this protein. A recent study has shown that CHI3L1 binds to IL-13Rα2, which plays a critical role in its effector responses [4]. It has been also reported that IL-13Rα2 signaling requires a scaffold protein, FAM120A, to activate the FAK and PI3K pathways in colon cancer metastasis [23]. Moreover, IL-13Rα2 utilizes TMEM219 in CHI3L1-induced signaling [19]. However, knockdown of FAM120A or TMEM219 in cells cannot completely suppress the response to CHI3L1 signaling. These data suggested the likely existence of an as-yet unknown functional receptor for CHI3L1.

Many lines of evidence indicated the tight relationship between CHI3L1 and HA, which together were considered as noninvasive markers of liver fibrosis [40–43]. As hyaluronan constitutes the likely preferred physiological ligand of CHI3L1 [28] and CD44 functions as a hyaluronan receptor, we speculated that CD44 may thus be a potential CHI3L1 receptor as well. In the present study, by using co-IP and co-location assay in two GC cell lines, we confirmed that CHI3L1 binds to CD44. CD44 is a cell surface adhesion receptor that is highly expressed in many cancers and has also been identified as a marker for several types of cancer stem cells [31, 44]. It has multiple ligands including proteoglycan 4, hyaluronate, laminin, collagen, fibronectin, osteopontin and galectin-9 [31, 39, 45]. Accumulating evidence demonstrates that CD44 acts as a multidomain signaling platform that integrates extracellular matrix cues with growth factor and cytokine signals [46]. The close interactions between CD44 and its selected binding partners play a pivotal role in coordinating “cross-talk” among various intracellular signaling pathways leading to the concomitant onset of multiple functions such as tumor cell proliferation and invasion [47]. Recently, CD44 was even shown to function in Wnt signaling by regulating LRP6 localization and activation [48]. Notably, our data show that CD44 also interacts with IL-13Rα2, which may account for the function of IL-13Rα2 in cancer metastasis. This allows for the hypothesis that IL-13Rα2 and TMEM219 behave similar to the α and β dimers of the T cell antigen receptor (TCR), which also have short intracytoplasmic tails and utilize other surface molecules such as CD3 to transmit signals [19, 49].

Next, we demonstrated that the activation of MAPK/Erk, Akt/PKB, and Wnt/β-catenin signaling by CHI3L1 was completely blocked by a CD44 neutralizing antibody. These data provide a logical connection to the known function of CD44 as a key regulator of tumor metastasis. In addition, our results indicated that the CHI3L1/CD44 axis serves as the critical mediator of GC cell colony formation, migration and invasion based on CD44 knockdown and function-blocking assays. These data provide strong evidence that the CHI3L1/CD44 pathway governs GC metastasis.

Activation of the β-catenin pathway has been reported as an early initiating event in gastric tumorigenesis [50]. Its activity depends on the accumulation and translocation of β-catenin to the nucleus, which is one of the hallmarks for the initiation of tumorigenesis in a variety of human cancers [51]. The Akt pathway can control nuclear localization of the Wnt signaling effector β-catenin through Akt-mediated phosphorylation of β-catenin at Ser552, resulting in a nuclear-localized form [52]. In the present study, we demonstrated, for the first time, that CHI3L1 also activates the Wnt/β-catenin signaling pathway by phosphorylating β-catenin at both Ser552 and Ser675, which in turn induces β-catenin accumulation in the nucleus and increases its transcriptional activity. In particular, the nuclear translocation of β-catenin involves the process of EMT [53]. Here, we demonstrated that CHI3L1 exerts its invasive function by enhancing the expression of vimentin and the EMT-inducing transcription factor snail. Additionally, our data show that CHI3L1 regulated migration and invasion of GC cells through synergistic activation of Erk, Akt, and Wnt/β-catenin signaling pathways (Additional file 2: Figure S1). Consequently, these promote cancer cell motility and invasion. Thus, it is reasonable to conclude that CHI3L1 expression may serve as a marker for GC malignancy. It is noteworthy that CHI3L1 is expressed by a variety of cells including neutrophils, macrophages, fibroblasts, vascular smooth cells, endothelial cells, and tumor cells [4]. Therefore, immune, inflammatory or stromal cells in the tumor microenvironment may also affect the biological behavior of tumor cells by secreting CHI3L1.

Previous studies have shown that IL-13Rα2 induces the expression of TGF-β [54, 55]. In many late-stage tumors, TGF-β activates the cellular EMT program that confers traits associated with high-grade malignancy on cancer cells [29, 30, 53]. As CD44 interacts with and enhances TGF-βRI activity [38, 39], our results suggest that the CHI3L1/CD44/IL-13Rα2 axis regulates the downstream effectors of driver oncogenes that contribute to GC survival, invasion, and metastasis.

CD44 is a ubiquitous transmembrane glycoprotein comprising a family of isoforms that are generated through alternative splicing of 10 variant exons (v1–v10) in the extracellular domain. The role of the CD44 family members in tumor progression and metastasis is most likely linked to the function of the various isoforms as signaling hubs [56]. It has been reported that the HS-modified CD44v3 isoform is necessary for hepatocellular carcinoma cells metastasis [57]. In addition, higher levels of CD44v3 were also observed in head and neck squamous cell carcinoma and therefore considered to enhance tumor cell migration [58]. Notably, inhibition of CD44v3 and CD44v6 function by copolymers carrying multiple copies of their targeted peptides blocks tumor invasion and metastatic colonization [59]. The peptide inhibitors of CD44v6 isoforms block tumor growth and metastasis in several independent models of pancreatic cancer [60]. However, in the present study, we show that CHI3L1 specifically binds to CD44v3 but not CD44v6 or CD44s, which indicated that the CD44v3 isoform in particular plays a critical role in CHI3L1 signaling.

CHI3L1 has been regarded as a potential diagnostic criteria and therapeutic targeting [61, 62]. Blockade of CHI3L1 activity or expression suppressed tumor vasculature in glioblastoma xenografted animals [9]. A CHI3L1-neutralizing antibody restrained tumor growth, angiogenesis, and progression [63]. Moreover, the conjunction therapy with the CHI3L1-neutralizing antibody and ionizing irradiation synergistically inhibited tumor vascularization and progression [64]. Notably, silencing of Chi3l1 expression in the lung using peptide-siRNA complex efficiently reduced mouse melanoma lung metastasis with enhanced Th1 and CTL responses [26]. Together, these findings suggest that CHI3L1 could be a therapeutic target to inhibit tumor progression and enhance anti-tumor immunity.

## Conclusions

In summary, our study demonstrated that CHI3L1 positively correlates with GC invasion depth, lymph node status, and tumor staging. Mechanically, CHI3L1 interacts with CD44v3 and activates Erk and Akt signaling, and notably triggers the β-catenin pathway by phosphorylating β-catenin at Ser552 and Ser675, which promote EMT in GC cells. Our data have thus identified the CHI3L1/CD44 axis as a vital pathway and potential therapeutic target in GC metastasis.

## Additional files

Additional file 1: **Table S1.** List of antibodies used in the different applications. (DOC 50 kb)

Additional file 2: **Figure S1.** CHI3L1 promotes tumor cells invasion through synergistic activation of the Erk, Akt, and Wnt/β-catenin signaling pathways. Invasion ability was determined in AGS or MGC803 cells after treatment with Erk inhibitor U0126 (10 μM), Akt inhibitor LY294002 (10 μM), or Wnt/β-catenin inhibitor ICG001 (10 μM). The combinations were subjected in a transwell invasion assay as indicated. Data are presented as mean ± SEM. *p < 0.05; *p* > 0.05, no significance (n.s) as determined by Student’s *t* test. (TIF 4878 kb)

## Abbreviations

BLI: Bio-layer interferometry
BMDM: Bone marrow derived macrophage
CHI3L1: Chitinase 3-like 1
ECD: Extracellular domain
ELISA: Enzyme linked immunosorbent assay
EMT: Epithelial-to-mesenchymal transition
Erk: Extracellular regulated kinase
GC: Gastric cancer
HA: Hyaluronic acid
HS: Heparan sulfate
IHC: Immunohistochemistry
IL-13Rα2: Interleukin-13 receptor alpha 2
IP: Immunoprecipitation
MAPK: Mitogen activated protein kinase
TGF-β1: Transforming growth factor β1
