# Supplementary material for: Chitinase 3-like 1-CD44 interaction promotes metastasis and epithelial-to-mesenchymal transition through β-catenin/Erk/Akt signaling in gastric cancer
Source: J Exp Clin Cancer Res. 2018 Aug 30;37:208. doi: 10.1186/s13046-018-0876-2 (PMC6117920; doi:10.1186/s13046-018-0876-2)
Supplement: Supplementary file 1 — Table S1. List of antibodies used in the different applications. (DOC 50 kb) [file 13046_2018_876_MOESM1_ESM.doc]

**Table S1 List of antibodies used in the different applications**

| Antibodies | Source | Identifier |
| --- | --- | --- |
| CHI3L1 | Abcam | Cat. #ab180569 |
| CHI3L1 | Abcam | Cat. # ab77528 |
| CHI3L1 (clone A-10) | Santa Cruz Biotechnology | Cat. #sc-393590 |
| CHI3L1 (clone D-11) | Santa Cruz Biotechnology | Cat. # sc-393494 |
| IL-13Rα2 (clone 2K8) | Santa Cruz Biotechnology | Cat. #sc-134363 |
| IL-13Rα2 (clone 2E10) | Abcam | Cat. #ab55275 |
| IL-13Rα2 | R&D Systems | Cat. # AF146 |
| CD44 (clone IM-7) | eBioscience | Cat. #16-0441-82 |
| CD44-FITC (clone IM-7) | eBioscience | Cat. # 11-0441-82 |
| Active-β-catenin (clone 8E7) | Millipore | Cat. #05-665 |
| phospho-β-catenin(Ser552) ( clone D8E11) | Cell Signaling Technology | Cat. #5651 |
| phospho-β-catenin(Ser675) ( clone D2F1) | Cell Signaling Technology | Cat. #4176 |
| Vimentin (clone D21H3) | Cell Signaling Technology | Cat. #5741 |
| Snail (clone C15D3) | Cell Signaling Technology | Cat. #3879 |
| Phospho-Akt (Ser473) (clone D9E) | Cell Signaling Technology | Cat. #4060 |
| Akt (pan) (clone C67E7) | Cell Signaling Technology | Cat. #4691 |
| Phospho-p44/42 MAPK (Erk1/2) (clone E10) | Cell Signaling Technology | Cat. #9106 |
| p44/42 MAPK (Erk1/2) | Cell Signaling Technology | Cat. #9102 |
| Donkey anti-Rabbit IgG (H+L) Highly Cross-Adsorbed Secondary Antibody, Alexa Fluor 568 | Thermo Fisher Scientific | Cat. # A10042 |
| Donkey anti-Mouse IgG (H+L) Highly Cross-Adsorbed Secondary Antibody, Alexa Fluor 568 | Thermo Fisher Scientific | Cat. # A10037 |
