# Supplementary figures and images for: Chitinase 3-like 1-CD44 interaction promotes metastasis and epithelial-to-mesenchymal transition through β-catenin/Erk/Akt signaling in gastric cancer
Source: J Exp Clin Cancer Res. 2018 Aug 30;37:208. doi: 10.1186/s13046-018-0876-2 (PMC6117920; doi:10.1186/s13046-018-0876-2)

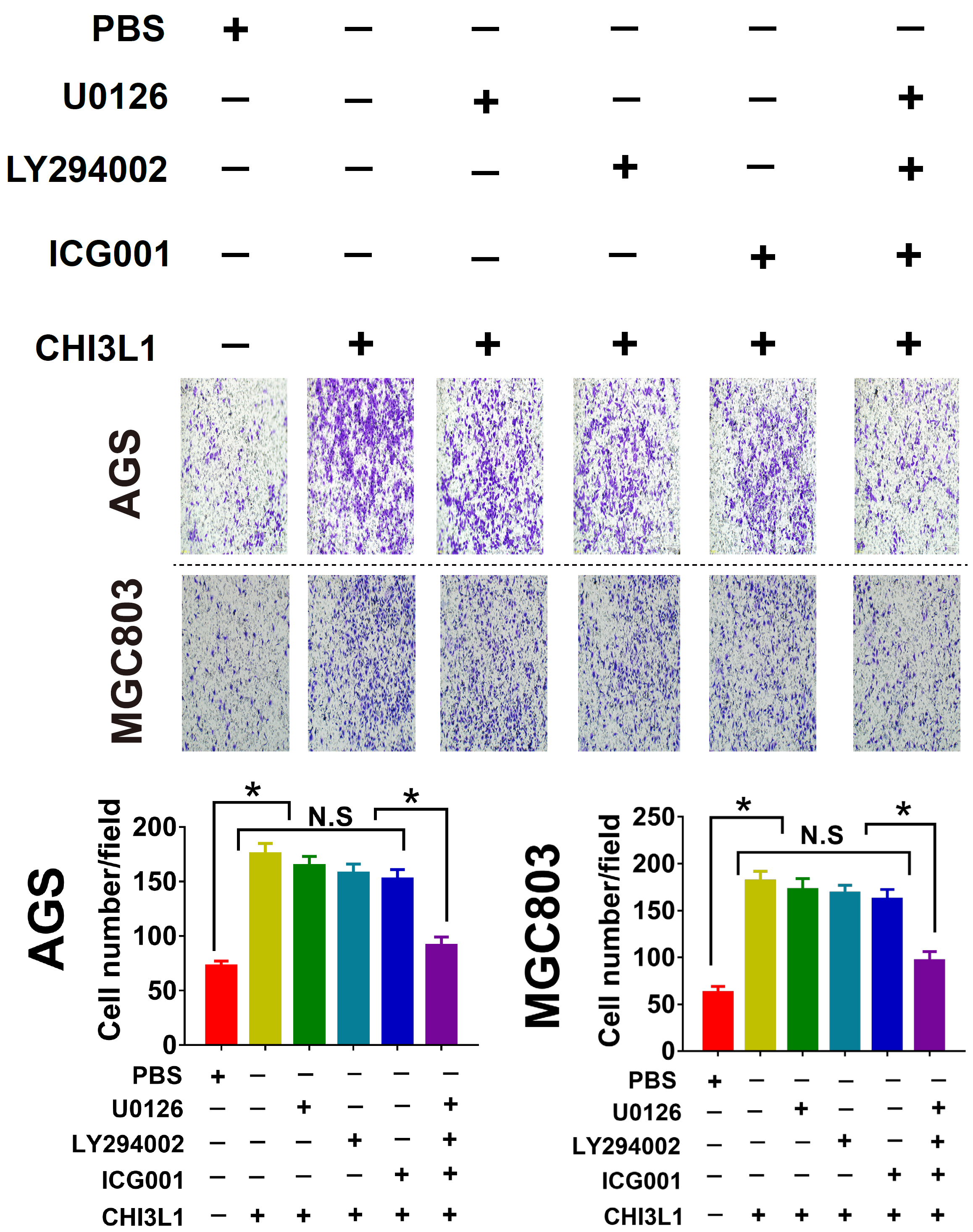

Supplement: Supplementary file 2 — Figure S1. CHI3L1 promotes tumor cells invasion through synergistic activation of the Erk, Akt, and Wnt/β-catenin signaling pathways. Invasion ability was determined in AGS or MGC803 cells after treatment with Erk inhibitor U0126 (10 μM), Akt inhibitor LY294002 (10 μM), or Wnt/β-catenin inhibitor ICG001 (10 μM). The combinations were subjected in a transwell invasion assay as indicated. Data are presented as mean ± SEM. *p < 0.05; p > 0.05, no significance (n.s) as determined by Student’s t test. (TIF 4878 kb) [file 13046_2018_876_MOESM2_ESM.tif]
